# Satellite glial cells modulate cholinergic transmission between sympathetic neurons

**DOI:** 10.1371/journal.pone.0218643

**Published:** 2020-02-04

**Authors:** Joana Enes, Marián Haburčák, Surbhi Sona, Nega Gerard, Alexander C. Mitchell, Wenqi Fu, Susan J. Birren

**Affiliations:** 1 Department of Biology, Brandeis University, Waltham, MA, United States of America; 2 Volen National Center for Complex Systems, Brandeis University, Waltham, MA, United States of America; University Hospital Wurzburg, GERMANY

## Abstract

Postganglionic sympathetic neurons and satellite glial cells are the two major cell types of the peripheral sympathetic ganglia. Sympathetic neurons project to and provide neural control of peripheral organs and have been implicated in human disorders ranging from cardiovascular disease to peripheral neuropathies. Here we show that satellite glia regulate synaptic activity of cultured postnatal sympathetic neurons, providing evidence for local ganglionic control of sympathetic drive. In addition to modulating neuron-to-neuron cholinergic neurotransmission, satellite glia promote synapse formation and contribute to neuronal survival. Examination of the cellular architecture of the rat sympathetic ganglia *in vivo* shows this regulation of neuronal properties takes place during a developmental period in which neuronal morphology and density are actively changing and satellite glia enwrap sympathetic neuronal somata. Cultured satellite glia make and release factors that promote neuronal activity and that can partially rescue the neurons from cell death following nerve growth factor deprivation. Thus, satellite glia play an early and ongoing role within the postnatal sympathetic ganglia, expanding our understanding of the contributions of local and target-derived factors in the regulation of sympathetic neuron function.

## Introduction

Glial cells, once thought of as neuron support cells, are now recognized as active players in the formation and function of normal brain circuitry [1, 2]. Astrocytes, the most abundant glial cell type in the brain, regulate many properties of neuronal circuits such as neuronal excitability, synaptic transmission and plasticity [3–5]. Their role at central nervous system (CNS) synapses has been the focus of a number of studies in the past two decades, showing that astrocytes control the formation [6–8], maturation [9], function [10, 11] and refinement [12] of synapses. These functions are mediated by various secreted as well as contact-dependent signals [11, 13, 14]. In addition to their role in the development and function of neuronal circuits [15], glia also play an important role in neurological disease, with astrocytes responding and contributing to human conditions ranging from developmental to degenerative disorders and traumatic lesions [16, 17].

In contrast to the wealth of information available on the roles of CNS astroglia, we have only a limited understanding of the satellite glia found in peripheral ganglia. This is particularly true for the sympathetic nervous system, which innervates most internal organs and regulates their function. A basal level of sympathetic activity, or sympathetic tone, together with opposing activity from the parasympathetic nervous system, ensures bodily homeostasis. Sympathetic tone may rise on a short timescale in response to a physiological demand (for example, exercise or stress) [18, 19], or over a long timescale, in a sustained manner, under pathological conditions such as hypertension and chronic heart disease [20, 21]. Sympathetic tone is initially set by neurons present in the brain and spinal cord [22], with the sympathetic ganglionic neurons acting as the final regulatory element determining the output of the sympathetic circuit.

A striking anatomical feature of the sympathetic ganglion is the presence of satellite glia that form an envelope around individual ganglionic neuronal somata and cover synapses [23]. This is in contrast to the CNS where individual astrocytes are in contact with multiple neurons [24]. Sympathetic and sensory satellite glia share some cellular and molecular features with astrocytes, including expression of neurotransmitter receptors and the formation of a glia network via gap junctions [25]. However, the functional role of peripheral glia, in particular sympathetic satellite glia, remains to be fully described. While embryonic cell culture experiments have shown that glioblasts interact with neuroblasts to promote neuronal differentiation, dendrite development, and ion channel expression, [26–29], less is known about how developing neurons and glia interact in the postnatal animal and how those interactions regulate the functional maturation of the sympathetic system.

Recent studies using genetic manipulations of adult sympathetic satellite glia have implicated these cells in the regulation of target organ function by demonstrating that selective activation of Gq-GPCR (G protein-coupled receptor) signaling in peripheral glia leads to the modulation of cardiac properties in adult mice [30, 31]. These effects are mediated through postganglionic sympathetic innervation of the heart, raising the possibility that activated glia influence the activity state of sympathetic neurons within the ganglia. This idea is supported by the finding that ganglionic cells can alter the short-term plasticity of single sympathetic neurons cultured in isolated conditions [32]. Yet, while these studies point to satellite glia as important potential regulators of sympathetic output, their effects on the formation and function of cholinergic synapses in the developing sympathetic system remains to be determined.

**Fig 1:**
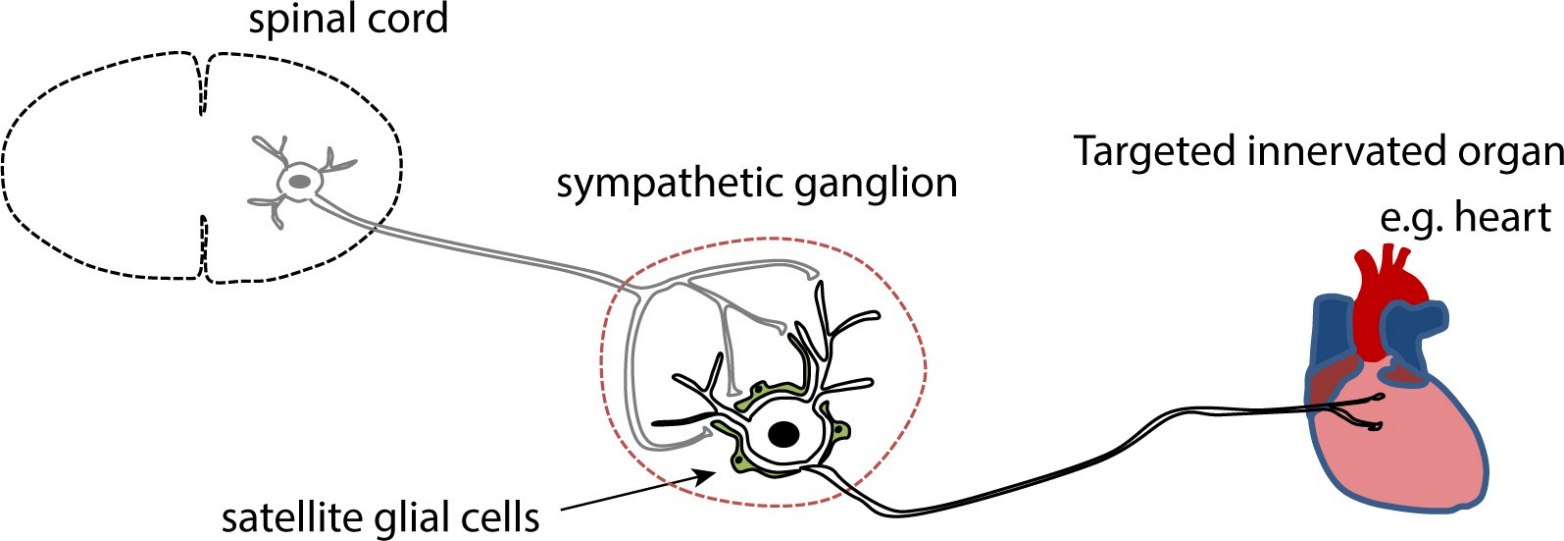
Schematic of the peripheral sympathetic-cardiac circuit. Within the sympathetic ganglia, pre-synaptic inputs from spinal cord preganglionic neurons form cholinergic synapses onto postganglionic sympathetic neurons, and satellite glial cells in the ganglia enwrap neuronal soma. The postganglionic neurons project to peripheral targets including the heart.

Here we show that satellite glia regulate the strength and firing rate of spontaneous synaptic transmission between co-cultured sympathetic neurons. We found that satellite glia promote the formation of synaptic sites with a specific effect on the formation of presynaptic structures. These *in vitro* findings are consistent with the developing architecture of the sympathetic ganglia *in vivo*, where satellite glia surround sympathetic neuronal soma during a period of postnatal neuronal maturation. We also explored the influence of these glia on sympathetic neuron survival and found that satellite glia promote the survival of cultured sympathetic neurons via a nerve growth factor (NGF)-dependent mechanism, suggesting glial contributions to neuron survival that are independent of known target-dependent survival pathways. These experiments provide insight into the development and modulation of sympathetic tone at the sympathetic-cardiac circuit’s last neuron-to-neuron synapse (see Fig 1).

## Materials and methods

### Cell culture

All experimental procedures involving animals were approved by the Brandeis Institutional Animal Care and Use Committee. Superior cervical sympathetic ganglia (SCG) were dissected from P1-P3 Sprague-Dawley (SD) rats, and either processed for immunohistochemistry or used to generate primary cultures of sympathetic neurons and glia. To generate primary cultures, isolated ganglia were de-sheathed, and incubated at 37° C for 1 hour in minimum essential medium (Gibco BRL, Invitrogen, Carlsbad, CA, USA) containing 350 units/ml collagenase type I (Worthington Biochemical Corporation, Lakewood, NJ, USA) and 5.5 units/ml dispase (Gibco BRL, Invitrogen, Carlsbad, CA, USA). Following enzymatic digestion, the cells were dissociated by passing repeatedly through fire-polished glass pipettes, and pre-plated on uncoated plastic tissue culture dishes for 1 hour at 37° C to remove non-neuronal flat cells. The less adherent cells, which consisted of aggregates of neurons and satellite glia, were then rinsed off the dishes, and plated at a density of 10,000 cells per dish on glass-bottomed plates (MatTek Corporation, Ashland, MA, USA) coated with collagen (50 μg/ml; BD Biosciences, Bedford, MA, USA) and mouse laminin (5 μg /ml; BD Biosciences, Bedford, MA). Cultures were maintained in modified L15CO_2_ medium [33, 34], supplemented with 10% fetal bovine serum (Omega Scientific, Tarzana, CA, USA and Gibco), 6 μg/ml dextrose, 2 mM glutamine (Invitrogen, Carlsbad, CA, USA), 100 U/ml penicillin & 100 μg/ml streptomycin (Invitrogen, Carlsbad, CA, USA), 1 μg/ml 6,7, dimethyl-5,6,7,8-tetrahydropterine (DMPH4, Calbiochem, San Diego, CA, USA), 5 μg/ml glutathione (Sigma, St. Louis, MO, USA) and 100 μg/ml l-ascorbic acid. Unless otherwise stated, mouse 2.5S NGF (5 ng/ml, BD Biosciences) was added to all cultures to support neuronal survival. Half of the media was exchanged with fresh NGF-containing growth medium three times weekly. To obtain glia-free neuronal cultures, cytosine arabinofuranoside (AraC, 1 μM, Sigma, St. Louis, MO, USA) was added to the cell culture media from day 1 to day 3 to inhibit glia cell division. To obtain neuron-glia co-cultures, AraC was withheld from the media, allowing satellite glial cells to proliferate rapidly, reaching 100% confluency at around 7–10 div (days in vitro). In some experiments we first used AraC to obtain glia-free neuron-alone cultures and satellite glial cells were re-plated on top of the neurons after 7 day of culture at approximately 100,000 glial cells per dish (NG[7]). These glia also formed a confluent layer by 10–14 div. Under all of these culture conditions, about 95% of the non-neuronal cells stained positive for S100β, a glial-cell marker.

### Electrophysiology

Neuronal whole-cell patch-clamp recordings were made using an Axopatch 200B amplifier (Axon Instruments, Union City, CA, USA). Extracellular solution contained, in mM: NaCl 150, KCl 3, MgCl_2_ 2, HEPES 10, CaCl_2_ 2 and D-glucose 11; pH 7.4 and adjusted to 320 mOsm with sucrose. Patch pipettes had resistances of 2–4 MΩ and were filled with internal solution containing, in mM: K gluconate 100, KCl 30, MgSO_4_ 1, EGTA 0.5, HEPES 10, K_2_ATP 2, NaGTP 0.3, Tris phosphocreatine 10; pH 7.2 and adjusted to 290 mOsmol with sucrose. All recordings were made at 33–35° C using a QE-1 heated culture dish platform (Warner Instruments Inc., Hamden, CT, USA). Data were acquired with pClamp 8 software suite and digitized at 10 kHz and low-pass filtered at 2 kHz. Electrophysiological responses were analyzed using built-in functions in MATLAB (The MathWorks, Inc., Natick, MA, USA).

Spontaneous activity was recorded for 5 minutes at a holding potential of -60 mV; cells were classified as silent if they showed fewer than 10 single events in the 5 min period. Total synaptic charge was defined as the area above the curve, i.e. the sum of all the current values above a threshold of 25 pA. Average synaptic charge corresponds to values calculated per 10 s duration. Values presented in plots are average membrane currents quantified as averaged synaptic charge normalized to 1 ms duration. Due to incomplete voltage clamp, we occasionally found cells that showed escaping action potentials identifiable based on an amplitude > 1 nA and duration < 7.5 ms. Those spikes were excluded from the quantification by cutting them off from the original trace and replacing them by interpolated values. Series resistance (R_s_) was monitored throughout recordings but not compensated. Cells were accepted for analysis only if they met the following criteria: a) resting Vm < -45 mV, b) R_series_ < 20 MΩ, c) R_input_ > 100 MΩ and not varying more than 20% of the initial value over the course of the recording.

Evoked activity was recorded in normal extracellular solution containing the nicotinic cholinergic antagonist hexamethonium bromide (100 μM; Sigma, St. Louis, MO, USA). A small dc current was injected to maintain membrane potential at -60 mV in between depolarizations. To examine the firing properties, incremental current pulses of 500 ms duration were injected into the cell. The average cell response was calculated from 3 consecutive trials.

### Immunocytochemistry

Cultured cells were fixed with 4% paraformaldehyde and stained for ß-tubulin class III with ms anti-Tuj-1 antibody (Covance; 1:2000), for the glial cell marker S100β with rb anti-S100 (Agilent Dako; 1:1000) and for nuclei with DAPI (4',6-Diamidino-2-Phenylindole Dihydrochloride; Invitrogen; 1:500). The antigen-antibody complex was visualized using the secondary antibodies dk anti-ms Rhodamine (1:500) and dk anti-rb FITC (1:500). Synaptic puncta were identified by the co-localization of pre-synaptic Vesicular Acetylcholine Transporter (VAChT) protein and the post-synaptic Shank protein in MAP2 stained neurons using rb anti-VAChT (Sigma Aldrich; 1:1000), ms anti-shank (Neuromab; 1:200) and ck anti-MAP2 (Chemicon, 1:1000) primary antibodies in conjugation with 1:500 diluted gt anti-rb Alexa 647, gt anti-ms Alexa 488 and gt anti-ck Alexa 568 secondary antibodies (Invitrogen).

### Synapse quantification

Sixteen bit images of 15–30 isolated neurons across 2 coverslips per condition from 3 independent cultures were acquired using a Leica DM6000 Confocal microscope under a 63x oil objective at zoom 3 and 1024x1024 resolution. Images were acquired sequentially under identical settings of laser strength, detector gain and detector offset across all conditions within each culture. These settings were chosen to exclude signal saturation in each channel using Quick Lookup Tables (QLUT) available in the Leica image acquisition software. The maximum intensity projection of each image was then analyzed using Puncta Analyzer (an ImageJ plugin written by Barry Wark and available upon request from c.eroglu@cellbio.duke.edu). The number and size of synaptic puncta on SCG neuronal cell bodies and proximal dendrites (<50 μm) were quantified using identical threshold values for all cells in both conditions. The number of synaptic puncta was normalized to the MAP2-positive area. Size of synaptic puncta, defined as the total area of co-localized pre- and post-synaptic markers, was calculated by the ImageJ plugin.

### Immunohistochemistry

SD rats were euthanized by CO_2_ asphyxiation and SCG were dissected. The tissues were fixed for at least overnight in 4% paraformaldehyde (PFA) and then cryo-protected by incubating them in 30% sucrose solution at 4°C until the tissues sank. The tissues were placed in cryo-molds and embedded in O.C.T. (optimal cutting temperature) compound (Tissue-Tek O.C.T. Compound, Sakura Finetek, VWR, CA, USA) before freezing with dry ice. The tissues were cut into 10 μm, longitudinal sections in a cryostat (Leica CM3050, Buffalo Grove, IL, USA) and thaw mounted onto Fisherbrand^™^ ColorFrost^™^ Plus Microscope Slides.

The tissue sections were rehydrated in PBS before treatment with 10 mg/ml sodium borohydride solution and then incubated in 3% bovine serum albumin (BSA)/0.3% Triton X-100 solution for 1 hour. They were then incubated overnight with primary antibodies at the following concentrations: chicken anti-Microtubule Associated Protein 2 (MAP2) polyclonal antibody (Sigma-Aldrich, EMD Millipore, Darmstadt, Germany, AB5543, 1:1000) and rabbit anti-S100 calcium-binding protein B β-subunit (S100-β) polyclonal antibody (Agilent Dako, Santa Clara, CA, USA Z0311, 1:400). Following washing, they were incubated with donkey anti-chicken rhodamine and donkey anti-rabbit Alexa 488 secondary antibodies for 1.5 hours and then with 1 mg/ml 4’,6-diamidino-2-phenylindole (DAPI, Invitrogen Life Technologies) (1:20) for 15 mins. Subsequently, the slides were immersed briefly in distilled H_2_O and then mounted using 1:1 glycerol:PBS mounting solution. The sections were then imaged using the Zen software (Zeiss) on a Zeiss LSM 880 laser scanning confocal microscope.

### Cell density and morphology quantification

Three SCG sections per animal and 2–4 images per section were taken using the 561 nm, 488 nm and 405 nm lasers to excite the three fluorochromes: rhodamine, Alexa 488, and DAPI, respectively. Neurons in SCG sections were identified by MAP2 staining, glial cells by S100β staining, and nuclei by DAPI staining. The number of neurons was counted using the Cell Counter plug-in of the Fiji (SciJava Consortium) software. Using this method, neurons and glia were analyzed from three SCG and about 27 sections per condition and neuron size and cell density was determined from the maximal projection of the Z-stacks. Cells stained for both S100β and DAPI were identified as glial cells and the number and density of glial cells was calculated. Counting glial number is difficult due to the ensheathment properties of the cells. Briefly, we developed (and have made available on GitHub, see Enes2020), a MATLAB^™^ script to determine the number of glial nuclei in each image by calculating the total DAPI-positive-S100-positive double-labeled area and dividing that number by the median area of DAPI and S100-co-stained nuclear structures. This number represents the total number of glial nuclei associated with S100 staining. This was then divided by the total area of the field being counted to obtain a measure of glial density (defined as glial nuclei per mm^2^) in each section. The neuron soma size was measured by manually outlining MAP-2 stained neurons within a rectangular area of identical size and position in each image using Fiji software in sections stained for MAP2, S100β and DAPI.

### Preparation of glial cell-conditioned medium (GCM) and control medium (CM)

Ganglion cells were grown in serum- and NGF- containing media until confluence, about 7–9 days. The cells were then trypsinized and transferred to new 10 cm dishes. After 20 minutes, cells were washed 3 times with warm PBS to remove neurons, and cultured in serum-free NGF-free medium for 3 additional days. This glial cell-conditioned medium (GCM) was collected, centrifuged for 3 min to pellet cell debris, and concentrated using centrifugal concentrators (Sartorius, Bohemia, NY, USA) with a size cut-off filter of 5 kDa. By centrifuging at 1750xg for 90 minutes, GCM was concentrated to about 20x. GCM was then filtered through a 0.22 μm syringe filter and stored at -20°C. It was added to the cells at 1:3 ratio in fresh serum-containing media (final serum concentration of 7.5%). Control, unconditioned media (CM) was prepared by concentrating approximately 20x serum-free media using the same centrifugal concentrators, and added to the cells at a 1:3 ratio in fresh serum-containing media (final serum concentration is also 7.5%).

### NGF deprivation experiments

Cultures were either initially plated in the presence of 5 ng/ml NGF in serum-free medium, which was replaced after two days with NGF-free, serum-free medium, or plated in the absence of NGF. NGF-free cultures were treated with anti-NGF antibody (1:1000, final concentration 1 μg/ml, Santa Cruz Biotechnology, Dallas, TX, USA), or with CM or GCM (as described above) with or without the TrkA-IgG fusion protein [35] (2μg/ml, Genentech, South San Francisco, CA, USA). Cultures were imaged at either 3 or 12 days and surviving neurons were counted and analyzed as the percentage of the number of neurons present in the positive control condition, i.e. those grown in the presence of 5 ng/ml NGF in serum-containing medium.

### Statistics

Data from at least 3 independent sets of cell culture experiments and at least three animals for immunohistochemistry were pooled for analysis. Results are presented as mean ± s.e.m.; for immunocytochemistry and electrophysiology experiments n represents the number of neurons analyzed and for NGF deprivation experiments n represents the number of independent culture preparations; for immunohistochemistry n represents number of animals. Statistical analysis was done using SigmaStat or IBM SPSS software. t-tests or Mann-Whitney were used for comparisons. For multiple comparisons, ANOVA was used, followed by pairwise post hoc (Tukey’s HSD or Tukey-Kramer) comparisons.

## Results

### Satellite glia enhance spontaneous activity of cultured sympathetic neurons

**Fig 2:**
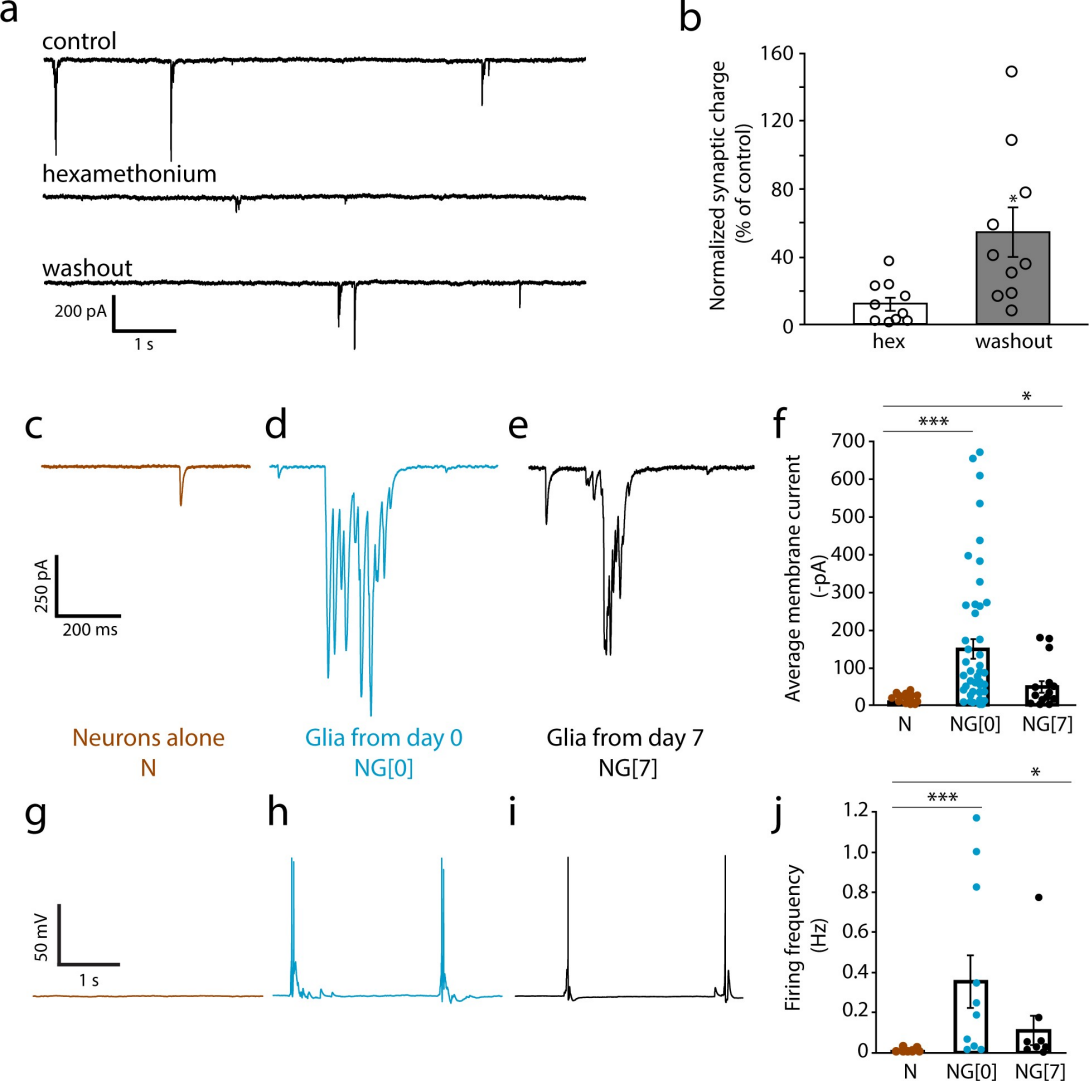
Satellite glial cells increase spontaneous activity of cultured sympathetic neurons. (a) Representative traces of spontaneous activity of neurons held in voltage-clamp at -60 mV, without hexamethonium, with 100 μM hexamethonium and after washout. (b) Average synaptic charge, normalized to control, for neurons treated with 100 μM hexamethonium (hex) and following washout (n = 10 cells per condition; paired t-test; *p<0.05). (c-j) Neurons were cultured for 14 days either alone (c, g) or in the presence of satellite glial cells starting from day 0 (d, h) or day 7 (e, i). NGF (5ng/ml) was included in all culture conditions to promote neuronal survival. (c-e) Representative voltage clamp traces showing that co-culture with satellite glial cells for the 14 days culture period, or for the last 7 days of the period increases current flow. (f) Quantification of synaptic activity. Total synaptic charge, defined as the area above the curve for neurons grown in the absence (Neurons alone, N) or presence of satellite glial cells for 14 days (NG[0]) or the last 7 days of the culture period (NG[7]), was quantified and average synaptic charge per 10 s duration was calculated. Plotted average membrane current values were quantified as averaged synaptic charge normalized to 1 ms duration. Therefore, the value of the average membrane current of e.g. -400 pA is equivalent to an average synaptic charge of 4 nC (n≥ 15 cells, Mann-Whitney U test, ***p<0.001, *p<0.05) (g-i) Representative current clamp traces showing that glial cells increase spontaneous firing in cultured sympathetic neurons. (j) Quantification of neuronal firing rate in the absence (N) or presence of satellite glial cells for 14 (NG[0]) or 7 (NG[7]) days. (n≥10 cells, Mann-Whitney U test ***p<0.001, *p<0.05). Bars represent mean ± s.e.m.; dots represent data for individual cells.

We examined whether satellite glia influenced the active properties of sympathetic neurons and whether the effects were at the level of intrinsic neuronal firing properties and/or synaptic activity. Sympathetic neurons form nicotinic cholinergic synapses onto each other when in culture for 2 weeks or longer [36], providing a valuable and often used [37–39] cell culture model to study ganglionic cholinergic transmission between spinal preganglionic neurons and the postganglionic sympathetic neurons. We confirmed the cholinergic nature of sympathetic transmission in the presence of satellite glia by recording neuronal activity before and after infusion of the nicotinic cholinergic antagonist hexamethonium bromide. Total activity was measured for 20 minutes and quantified under control (0–3 min), hexamethonium (6–9 min) and washout (16–19 min) conditions. Activity was reduced to about 10% of control after hexamethonium infusion, showing a partial recovery to 55% after 10 minutes of washout, indicative of the cholinergic nature of sympathetic transmission for neurons in the presence of satellite glia (Fig 2A and 2B).

We used this system to investigate the contribution of satellite glial cells to the development of sympathetic activity, measuring the spontaneous activity of sympathetic neurons cultured alone or in the presence of satellite glia. In the co-culture condition, neurons were grown with satellite glia for the full culture period (Glia from Day 0, NG[0]) as described in Methods. Spontaneous neuronal activity was recorded for 5 minutes for neurons in co-culture and neurons grown alone. We first assessed total current by recording in voltage clamp at a holding potential of -60 mV. We found that the presence of glia resulted in a strong increase (>18 fold) in the total charge of sympathetic neurons when compared to the neuron alone condition (Fig 2C, 2D and 2F). Bursts of activity were commonly observed in the presence of glia, but were absent in the neuron alone cultures.

Generation of the post-mitotic neuron-alone cultures requires the use of AraC to block glia proliferation. We asked if the use of AraC in these cultures contributed to the low level of activity in comparison to the neuron-glia co-cultures, which were grown in the absence of AraC. We recorded neuronal activity in cultures (named “glia from day 7”, NG[7]) in which AraC was initially added to prevent glial proliferation; at day 7, satellite glial cells grown in a separate dish were re-plated on top of the neurons at approximately 100,000 cells per dish. These glia also formed a confluent layer by 10–14 div. The presence of glia from day 7 also increased total activity by about 10 fold (Fig 2E and 2F), indicating that glia still exert their effect on spontaneous activity when added at a later time point in culture even when the neurons had been exposed to AraC treatment.

We next asked whether the increase in neuronal activity was accompanied by an increase in the frequency of action potential firing. We recorded from neurons in current clamp and measured spontaneous neuronal activity, finding that neurons cultured in the presence of glial cells fired more action potentials than neurons cultured alone (Fig 2G–2J). This increase in firing may underlie the occurrence of synchronous neurotransmitter release from pre-synaptic terminals and hence the bursts of activity seen in Fig 2D, 2E and 2F.

The effects of glial co-culture are independent of any effects of glia on neuronal survival, as these cultures were maintained in the presence of sufficient NGF to support survival (5 ng/ml) and we confirmed that there was no change in neuron number in cultures grown with glia. Overall, these results demonstrate that satellite glia derived from sympathetic ganglia increase the magnitude of neuronal inputs and the firing of cultured sympathetic neurons. Moreover, glia also exert their effects when added to the neuronal culture at a later stage (“glia from day 7”), suggesting that the effect is not dependent on early neurite extension and that glia act directly at synapses or at voltage-gated ion channels to enhance sympathetic activity.

### Excitable membrane properties of sympathetic neurons grown with satellite glia

We next investigated whether co-cultured glia affect neuronal firing properties in addition to synaptic properties. We examined the intrinsic membrane properties of neurons grown for 2–3 weeks alone or in the presence of satellite glia isolated from the same ganglia. We recorded from sympathetic neurons in whole cell current clamp and assessed resting membrane potential and input resistance (Table 1). There was no difference in neuronal input resistance between neurons grown in the presence or absence of glia. We observed a significant increase in neuronal resting membrane potential in the presence of glial cells.

**Table 1:** Neuronal characteristics of neurons co-cultured with or without glia.

Cultured conditions	Neurons alone	Neurons with glia
**Neuronal size (**μ**m**^**2**^**)** (soma with proximal dendrites)	580.1± 21.7 (n = 43)	740.6 ± 53.0** (n = 38)
**Resting membrane potential (mV)**	-53.3 ± 0.8 (n = 74)	-56.9 ± 1.0** (n = 49)
**Input resistance (MΩ)**	421.2 ± 33.2 (n = 37)	454.5 ± 67.3 (n = 36)
**Firing threshold (pA)**	83 ± 7.3 (n = 10)	118 ± 12.2 (n = 12)

All results are expressed as mean ± s.e.m.

**p<0.01 compared with controls (Neurons alone) using t-test.

**Fig 3:**
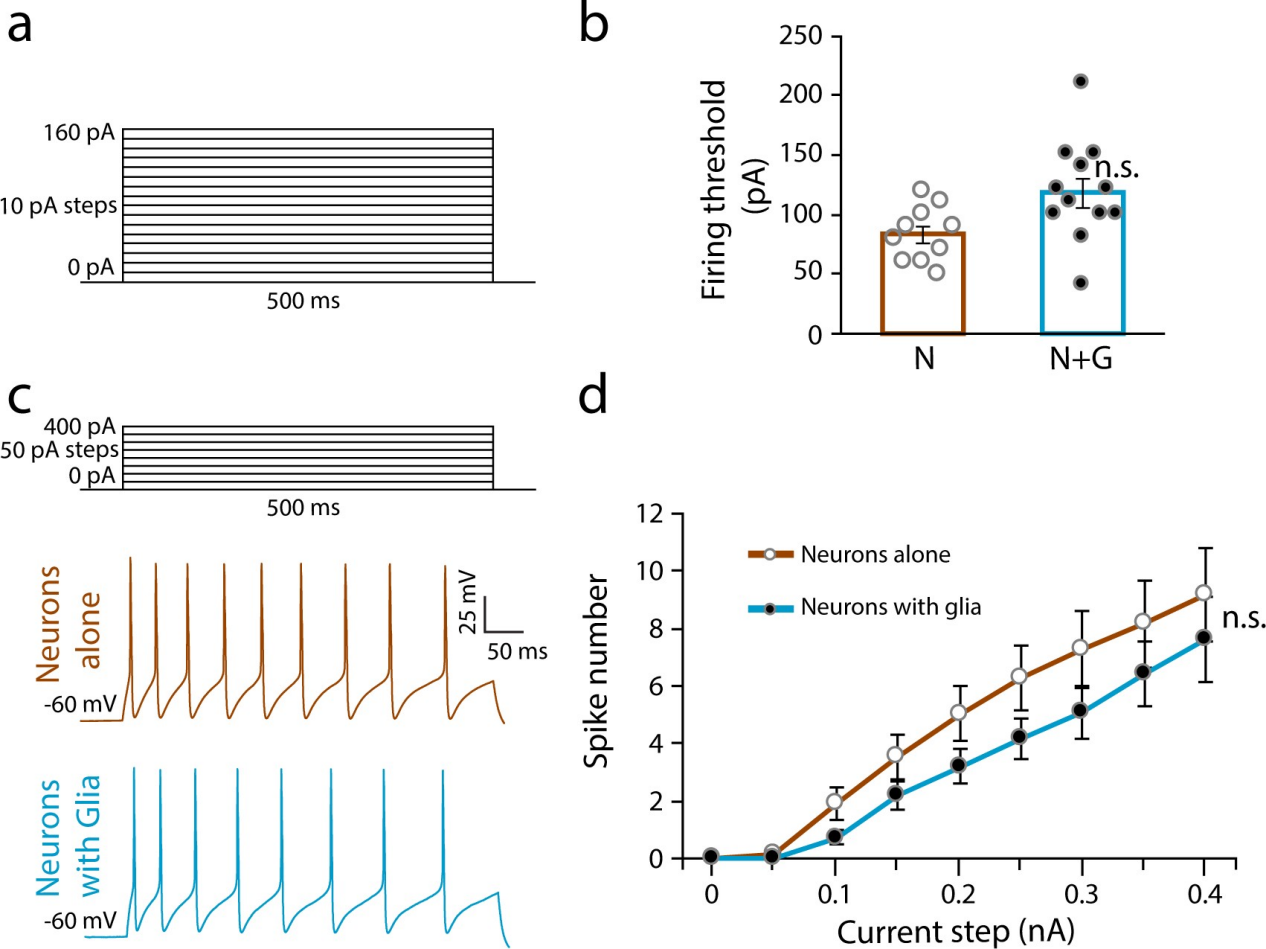
Satellite glial cells do not alter neuronal intrinsic excitability. (a) Illustration of the stimulus pattern applied to determine neuronal firing threshold. (b) Quantification of neuronal firing threshold between neurons alone (N) and neurons co-cultured with glia for 14 days (N+G) conditions. (Unpaired t-test, n ≥ 10 cells, n.s. not statistically different). (c) Illustration of the stimulus pattern applied to evoke action potential firing and representative neuronal traces in response to 400 pA current pulse for neurons grown for 14 days alone (brown, upper trace) or in the presence of glia (blue, lower trace). (d) Average number of action potentials evoked by current steps of increasing amplitude. (n≥16 cells, Mann-Whitney U test pairwise comparison for 400 pA current step, n.s. not statistically different). Results are represented as mean ± s.e.m.

We measured the effect of satellite glia on sympathetic intrinsic excitability by determining the neuronal firing in response to stimuli for neurons grown alone compared to neurons grown with glia. We analyzed the response of neurons to steps of depolarizing current in the presence of the cholinergic transmission blocker hexamethonium. Firing threshold, i.e. the minimum current needed to elicit an action potential, was determined by applying depolarizing currents steps in 10 pA increments (Fig 3A). We found a trend toward an increase in firing threshold in the presence of glia, although the difference was not statistically significant (Fig 3B). Next, we measured the number of spikes evoked by depolarizing current steps ranging from 0 to 400 pA (Fig 3C). There was a trend, which did not reach significance, towards a decrease in the number of APs fired by neurons cultured in the presence of glia in response to the current pulses (Fig 3D).

**Fig 4:**
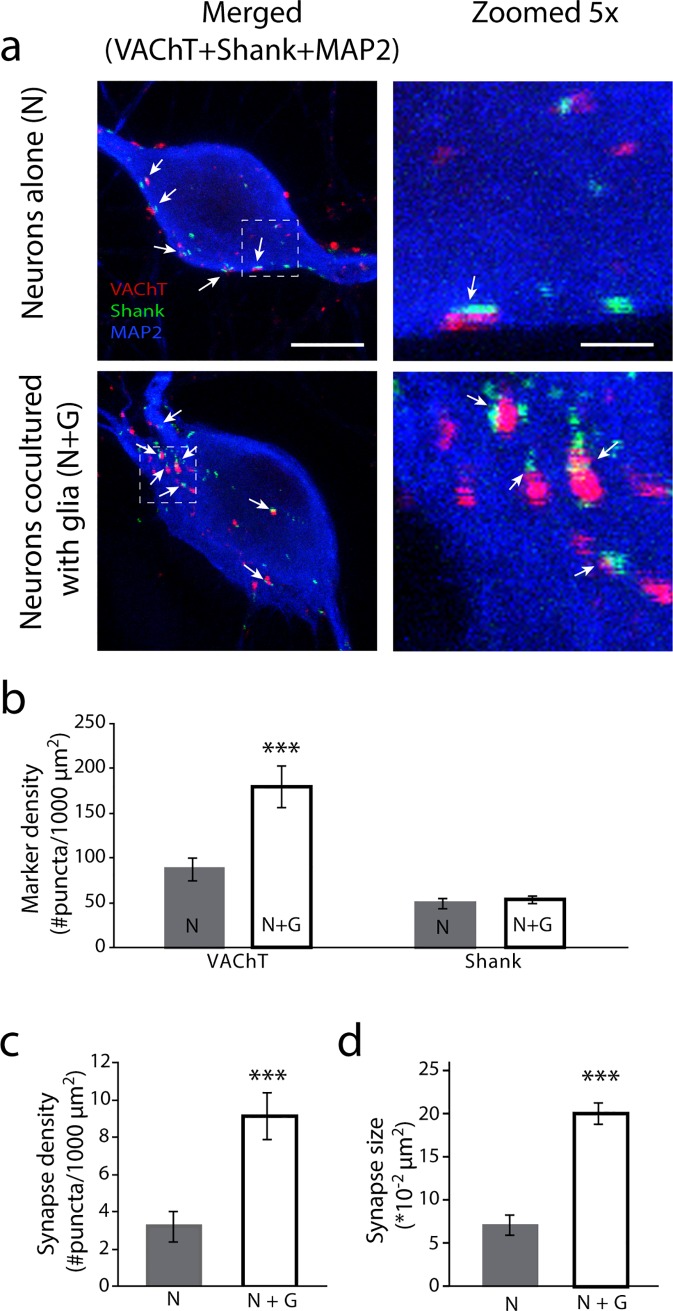
Satellite glial cells enhance cholinergic synapse formation. Neurons cultured in the absence (Neurons alone, N) or presence of satellite glial cells (N+G) were fixed, stained for synaptic markers, and analyzed by confocal microscopy. (a) Representative images of cells stained for the pre-synaptic marker VAChT (red), the post-synaptic marker Shank (green), and the dendritic marker MAP2 (blue). Boxed regions in left panels are magnified in the right panels to show colocalized puncta (arrows). Scale bar represents 10 μm in the left panels; 2 μm in the right panels. (b) Quantification of VAChT and Shank puncta on sympathetic neuronal soma and proximal dendrites showing a glia-dependent increase in the expression of VAChT, but not Shank-containing puncta (n ≥ 61 cells, unpaired t-test ***p<0.001 and n.s., respectively). Quantification of co-localized synaptic puncta density (c) and size (d) on neuronal soma and proximal dendrites showing that glia induce structural synapse formation (n ≥ 61 cells, unpaired t-test, ***p<0.001, **p<0.01, respectively).

We next asked whether glial cells had an effect on the development of synaptic sites. Soma and dendrites are important sites of synapse formation on peripheral sympathetic neurons [37, 40, 41]. We immunostained neurons using the vesicular acetylcholine transporter protein (VAChT) as a pre-synaptic marker, and the scaffold protein Shank (Shank) as a post-synaptic marker [42], and looked for their co-localization on cell bodies and proximal dendrites (Fig 4A). We quantified the number of pre-, post- and co-localized puncta and the size of co-localized puncta in cultures of neurons grown alone and in the presence of glial cells (Fig 4B–4D). Co-culture with glia increased the number (Fig 4C) as well as the area covered by colocalized puncta on sympathetic neurons (Fig 4D). There was a significant difference in the number of VAChT-positive puncta, but we observed no significant increase in the number of Shank-positive puncta (Fig 4B). This suggests a presynaptic effect of glia on the development of sympathetic synaptic sites, although additional postsynaptic mechanisms cannot be ruled out. While changes in the area covered by co-localized sites could reflect changes in synaptic protein clustering and do not necessarily reflect changes in synapse size, our results suggest that glia promote increased sympathetic activity by promoting structural synapse formation.

### Dynamic changes in the ganglionic structure of sympathetic neurons and satellite glia during the postnatal period *in vivo*

**Fig 5:**
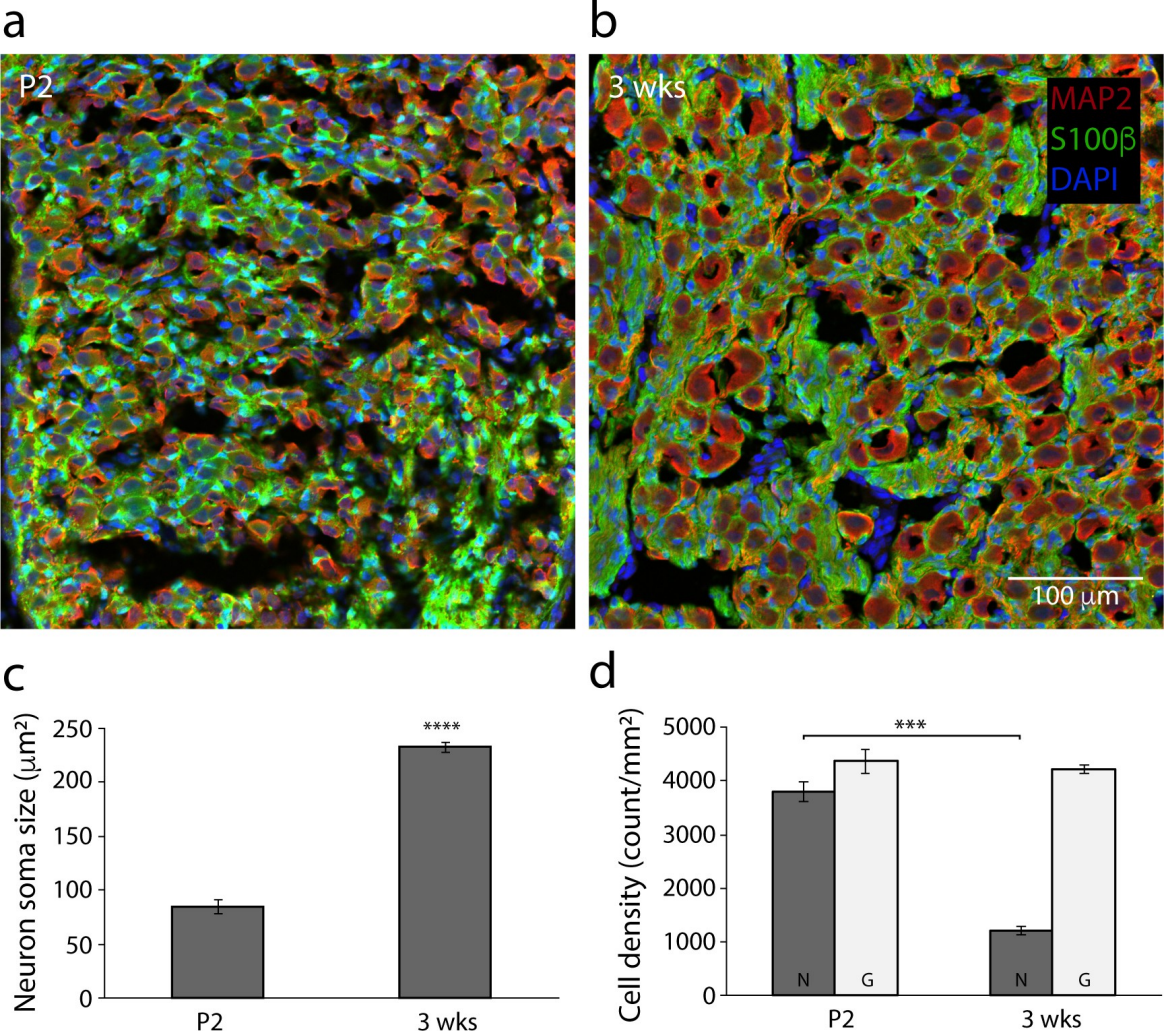
Postnatal development of neurons and satellite glia in the Superior Cervical Ganglion (SCG). (a-b) Representative confocal images of (a) P2 and (b) P21 SCG. Sympathetic neurons were stained for the neuron-specific marker MAP2 (red), satellite glial cells for the glial cell marker S100β (green) and cell nuclei using DAPI (blue). Scale bar = 100 μm. (c-d) Quantification of (c) neuron soma size, measured as average cell area in the section and (d) neuronal and glial cell densities from sections of P2 (n = 3; mean ± s.e.m.) and 3 wks (n = 3; mean ± s.e.m.). ***p<0.001, **p<0.01, *p<0.05 determined by ANOVA followed by pairwise post hoc (Tukey’s HSD) comparison test.

Our results show that satellite glia regulate synaptic and intrinsic properties of postnatal sympathetic neurons *in vitro*, raising the question of whether neurons and glia within the ganglia maintain the close interactions that could facilitate such regulation *in vivo* during this active developmental period [37]. We stained P2 and P21 SCG sections with MAP2 to identify neuronal soma and S100 to identify glia *in vivo* (Fig 5A and 5B) over a period similar to our *in vitro* cultures. We observed a close juxtaposition between neurons and glia at both time points, even as the morphology of the ganglia changed. Between P2 and P21, sympathetic neurons increased in size (Fig 5C) as the neurons make target contacts and are exposed to target derived signals that promote cellular hypertrophy [43]. As neurons increase in size the number of neurons per unit area decreases, while the density of satellite glial cells remains constant over the first three postnatal weeks (Fig 5D).

### Satellite glia exert their effect via released factors

**Fig 6:**
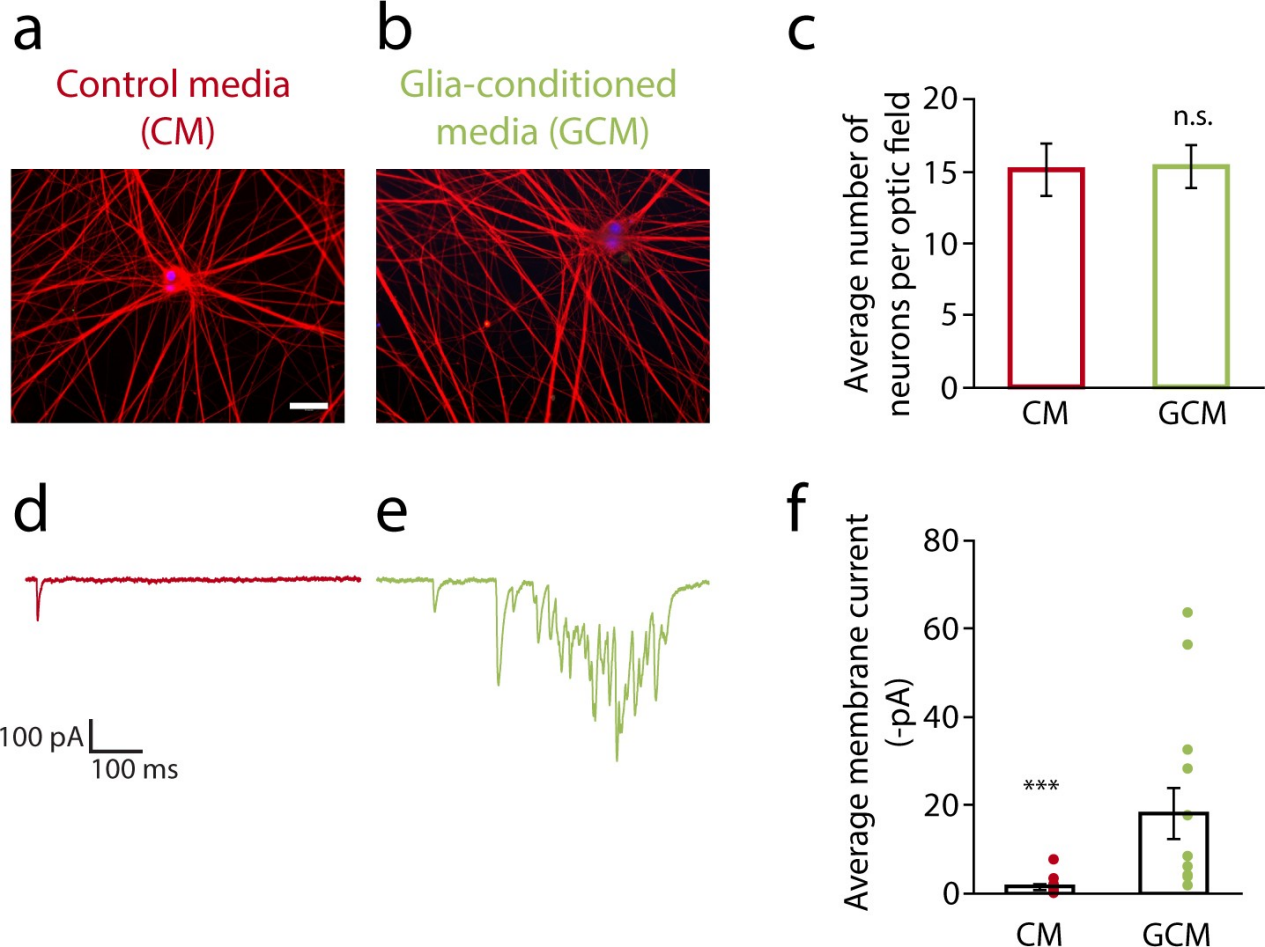
Glial cell-conditioned medium recapitulates the effect of satellite glial cells on cultured sympathetic neuron activity. (a-b) Neurons grown in control medium—CM (a) or glial cell-conditioned medium—GCM (b) for the last 7 days of the 14-day culture period. NGF (5ng/ml) was included in both culture conditions to promote neuronal survival. Cells were fixed and stained for Tuj-1 (neuronal marker, in red) and DAPI (nuclear staining, in blue). Scale bar represents 50 μm. (c) Neuronal cell number was not altered by the cell culture condition. (n = 6 independent cell culture experiments, unpaired t-test, n.s.). (d-e) Representative voltage-clamp traces of neurons cultured in CM (d) or GCM (e). (f) Quantification of average membrane current showing increased spontaneous activity in GCM (n ≥ 12 cells, non-parametric Mann-Whitney U test, ***p<0.001). Results are represented as mean ± s.e.m., dots represent data for individual cells.

The neuron and glial morphology seen in postnatal ganglia are consistent with the effect of glia on sympathetic synaptic activity being mediated by contact or by diffusible factors, or both. If diffusible factors play a role in this regulation, we would expect that glial cell-conditioned medium (GCM) would be sufficient to increase sympathetic neuron activity. We cultured glial cells until they reached confluency and allowed them to grow for an additional 3 days in serum-free medium before collecting the medium. The GCM was concentrated (see Methods) and added to sympathetic neurons that had been cultured alone for 7 days. Control medium (CM) from fresh serum-free medium (not conditioned by glial cells) was concentrated to the same level as the GCM and added to neurons following the same protocol as for GCM (Fig 6A and 6B). Following addition of GCM or CM neurons were cultured for an additional 7 days. We compared spontaneous activity of neurons cultured in the presence of CM or GCM at 14 div. The GCM did not affect neuronal survival, as neuron number was unaltered when compared to CM (Fig 6C). Culture in GCM resulted in a >13 fold increase in sympathetic activity (Fig 6D–6F), comparable to the effect observed in the presence of satellite glial cells in culture from day 7, the equivalent condition in the glial coculture experiments (see Fig 2E). These data indicate that factor(s) released by satellite glial cells increase sympathetic activity. While we cannot rule out additional effects of glial cell contact, it seems likely that released factors are the main modulators of sympathetic activity, at least in cell culture, since GCM fully mimics the effect of satellite glial cells cultured under the same condition (“glia from day 7”, NG[7]).

### Satellite glia support survival and hypertrophy of cultured sympathetic neurons

**Fig 7:**
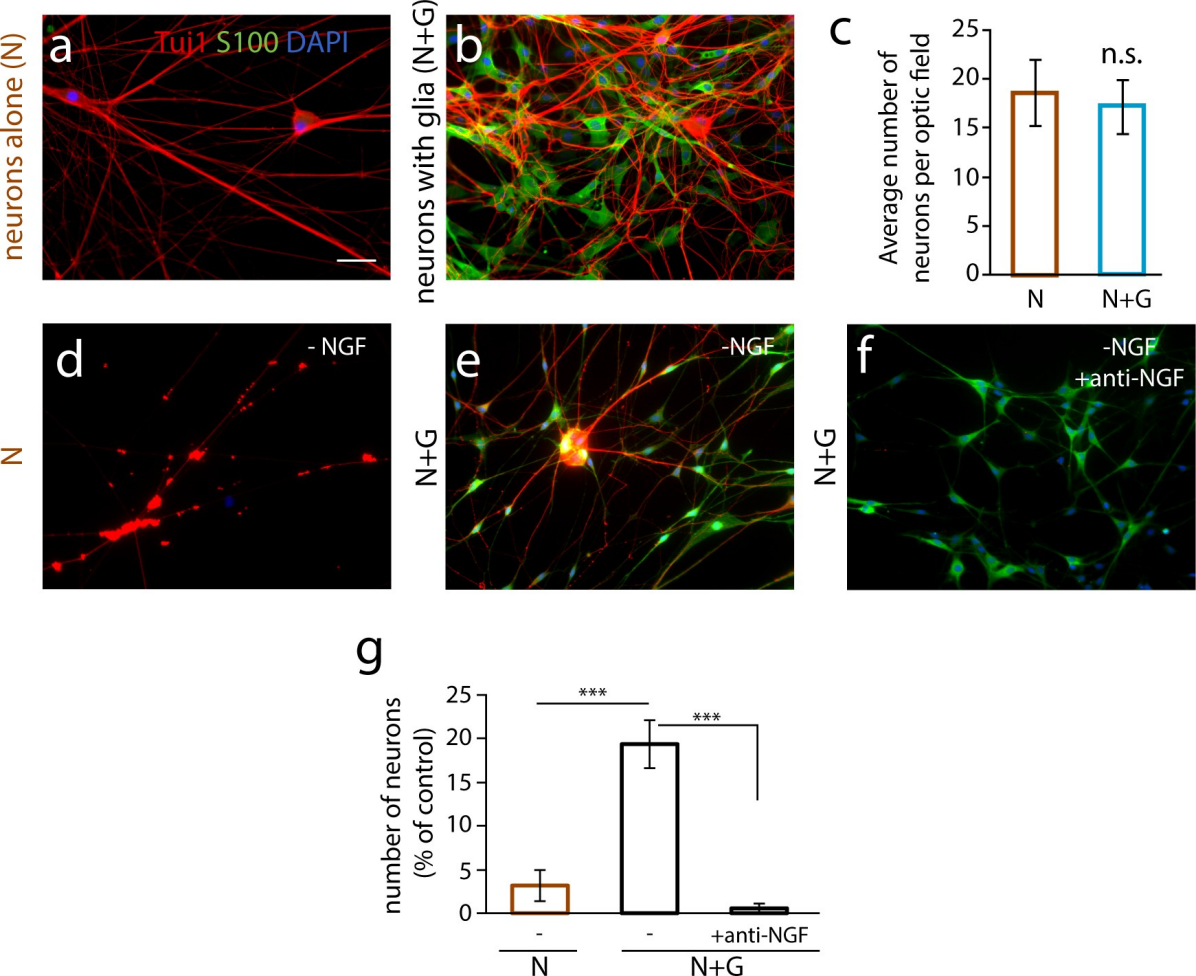
Satellite glial cells support neuronal survival. Satellite glial cells partly prevent sympathetic neuronal death upon NGF deprivation. (a-b) Establishment of sympathetic neuron-satellite glia co-cultures. Neurons (N) were cultured alone (a) or in the presence of satellite glia (b) in the presence of 5 ng/ml NGF in serum-containing medium. Cultures were fixed at 12 days *in vitro* (div) and stained for Tuj-1 (neuronal marker, in red), S100β (glial cell marker, in green) and DAPI (nuclear staining, in blue). Scale bar represents 50 μm. Under these growth conditions, glia did not alter the number of sympathetic neurons (c). (d) Neurons alone (N), (e) Neurons and glia (N+G), and (f) N+G with anti-NGF antibody (1:1000, final concentration 1 μg/ml). (g) Quantification of cell survival upon NGF deprivation. Data are shown as percent neuronal survival compared to comparable cultures (neurons alone or neurons + glia) grown in the presence of 5 ng/ml NGF in serum-free medium (n = 3 independent cell culture experiments, One-way ANOVA, ***p<0.001). All data are represented as mean ± s.e.m.

We asked if satellite glial cells provided neurotrophic support for sympathetic neuron development in neonatal cultures by determining whether co-cultured glia promoted neuronal hypertrophy and the survival of NGF-deprived sympathetic neurons. Neuronal soma areas were significantly larger in cultures containing co-cultured glia (Table 1), consistent with known effects of NGF on sympathetic hypertrophy [43]. We also asked if glial cells rescued neuronal survival following NGF withdrawal. Sympathetic neurons are normally cultured in the presence of 5 ng/ml NGF and, under these conditions of neurotrophic support, the addition of glia did not further support neuronal survival (Fig 7A–7C). We found that NGF deprivation lead to almost complete neuronal cell death (Fig 7D and 7G) of neurons grown alone, while co-culture with satellite glia resulted in a partial rescue of neuronal survival (Fig 7E and 7G). We next asked if the survival effects of co-cultured glia were due to glia-derived NGF. NGF-deprived co-cultures of sympathetic neurons and satellite glia were treated with an anti-NGF antibody to block endogenous NGF in the cultures (Fig 7F and 7G). The survival effect of glial co-culture was abrogated following the anti-NGF treatment, indicating that glial-produced neurotrophic factors can contribute to sympathetic neuron survival during the postnatal period.

**Fig 8:**
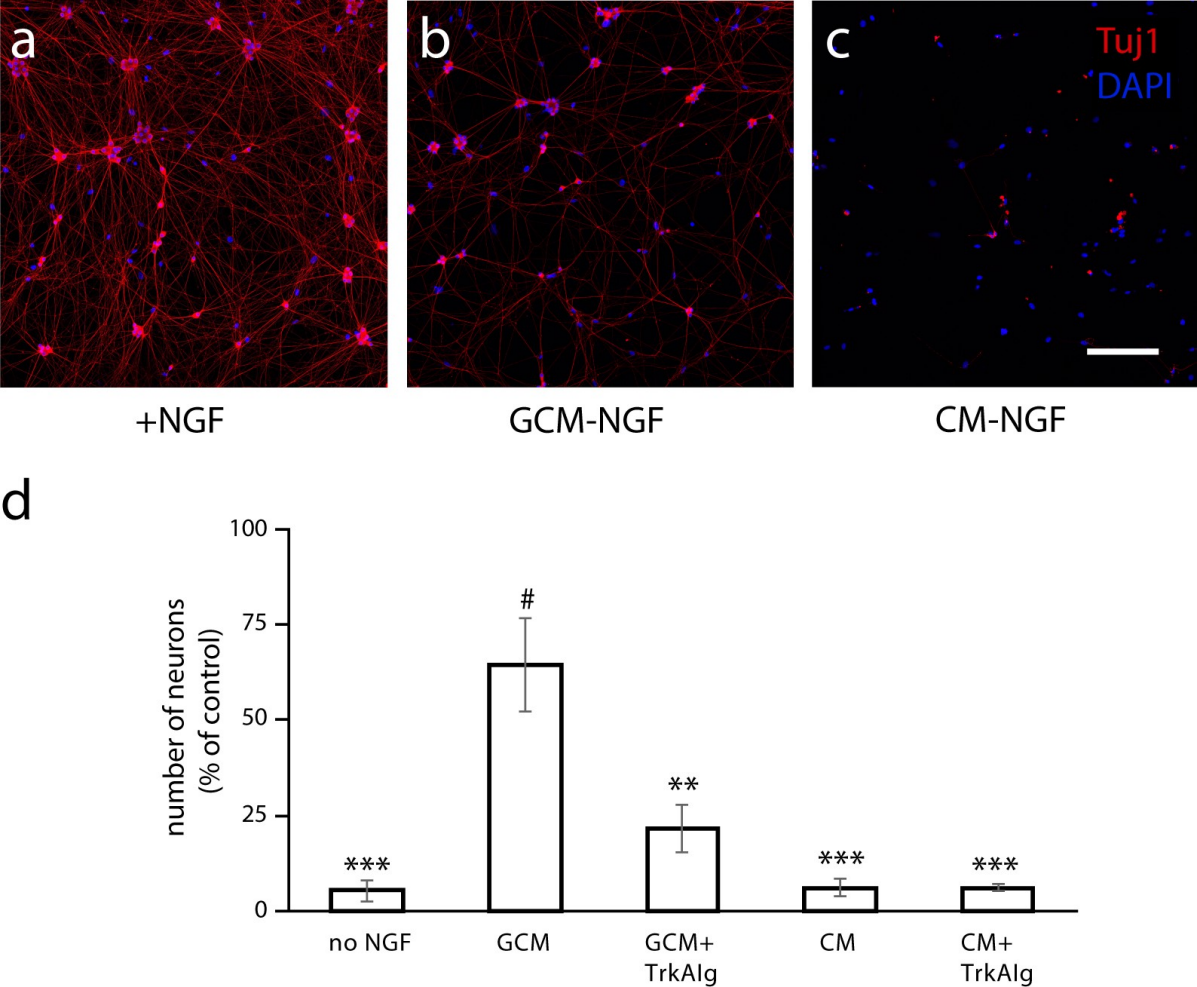
Satellite glial conditioned medium supports neuronal survival via an NGF-dependent mechanism. Representative images of neurons grown for 3 days in (a) 5 ng/ml NGF (+NGF), (b) glial conditioned medium without added NGF (GCM-NGF), and (c) control conditioned medium without added NGF (CM-NGF). Cultures were fixed at 3 div and stained with Tuj1 (red) an DAPI (blue). Scale bar: 200 μm. (d) Quantification of percent survival compared to the NGF condition for neurons cultured for 3 days in no NGF, GCM, GCM+TrkA-IgG, CM, CM+TrkA-IgG (n ≥ 4 independent cell culture experiments, **p<0.01, ***p<0.001, compared to GCM; ^#^p<0.001, compared to no NGF condition; ANOVA followed by pairwise post hoc (Tukey-Kramer) comparison test. All data are represented as mean ± s.e.m.). Note that the greater survival effect of GCM compared to the co-culture with the glial cells (Fig 7), is likely to reflect the analysis of the co-cultures at a later time point than the GCM-treated cultures.

Finally, we asked if the neurotrophic effects of glial co-culture could be recapitulated by GCM, as would be expected if the survival effects were due to the release of NGF or other survival factors by the glial cells. Neurons were grown in the presence of NGF, or in the absence of NGF with GCM or CM. GCM rescued neurons from NGF deprivation, while CM had no effect on blocking neuronal death in the absence of NGF (Fig 8A–8D). Cultures treated with GCM in the presence of a TrkA-IgG fusion protein [35, 44] to scavenge soluble NGF, showed reduced survival compared to neurons grown in GCM without added NGF (Fig 8B), further supporting a role for glial-produced NGF in the promotion neuronal survival.

## Discussion

We report a role for satellite glial cells in the establishment of mature sympathetic neuron structure and function within peripheral ganglia. Satellite glia potentiate sympathetic cholinergic synaptic activity and structural synapse formation in cultures of postnatal sympathetic neurons. These findings are consistent with the cellular architecture observed in SCG *in vivo*, where satellite glia enwrap neuronal cell bodies during a postnatal period of neuronal maturation. Satellite glia also contribute to the survival of cultured postnatal sympathetic neurons, an effect that is mediated by the release of neurotrophic factors, including NGF, a known modulator of sympathetic neuron activity [38, 45, 46]. This work defines sympathetic satellite glia as regulators of peripheral neuronal development and provides a new path for understanding mechanisms leading to heightened sympathetic tone.

The actions of satellite glial cells in regulating synapse formation and neuronal activity within the sympathetic ganglia share some common features with astrocytes in the central nervous system [1, 47]. While this illustrates a convergence in function between these two glial cell types of different embryonic origins, this work defines a new role for glia in the regulation of peripheral sympathetic neurons. Thus, while astrocytes regulate many aspects of excitatory glutamatergic and inhibitory GABAergic transmission in the CNS [13, 48–50], here we show that, in rat peripheral neurons, sympathetic satellite glia promote the development of spontaneous network activity at cholinergic synapses.

Outside of the mammalian CNS, glial regulation of cholinergic systems has been reported in *Lymnaea stagnalis*, where cholinergic neurons grown in the presence of glial cells have decreased postsynaptic responses to presynaptic stimulation [51]. Non-neuronal ganglionic cells also regulate short-term plasticity at sympathetic cholinergic autapses without an effect on synaptic development [32]. Earlier work suggested a role in synapse formation by showing that unidentified non-neuronal cells promoted evoked release of acetylcholine in mass cultures of sympathetic neurons [52]. Taken together with our findings of increased synapse number and spontaneous synaptic transmission in sympathetic neurons co-cultured with satellite glial cells, these studies show that glial modulation of cholinergic properties is characterized by system-specific properties. Our work shows that within the developing sympathetic system, glial cells release soluble factors that contribute to the development and dynamics of cholinergic circuits.

System-specific characteristics of glial modulation are also seen by comparing satellite glial actions in peripheral sensory and sympathetic ganglia [23]. Satellite glial cells of the sensory ganglia have been studied in the context of abnormal pain conditions and were found to contribute to neuronal hyperexcitability [53, 54]. In our study we analyzed intrinsic excitability and did not find a significant difference in sympathetic neurons cultured in the presence of satellite glia (Fig 3). This differential effect of satellite glia in sympathetic and sensory ganglia may reflect anatomical differences between these ganglia, since sensory ganglia do not receive inputs from central preganglionic neurons, and do not contain dendrites or synapses. It thus seems likely that glia affect different neuronal properties in each of these two peripheral ganglia, an idea that is supported by the synaptic effects and absence of changes in excitability in our cultures.

Our finding of glial regulation of cholinergic transmission and presynaptic protein expression suggests a regulatory circuit in which glial factors act to increase neuronal acetylcholine release, which may in turn act on the glial cells to modulate glial activity. This model is supported by recent work demonstrating changes in sympathetic satellite glial activity in response to glial muscarinic cholinergic receptor activation [55]. This cholinergic signaling resulted in an increase in glial calcium signaling, glial activation and electrical coupling between glial cells, suggesting that activity in neural circuits may be set by reciprocal signaling between neurons and their surrounding glia.

In the CNS, astrocyte cell function is also modulated by cholinergic signaling, resulting in glial regulation of glutamatergic or GABAergic neurotransmission. In the hippocampus, for instance, astrocytes act as a sensor for septal-derived acetylcholine associated with wakefulness, resulting in gating of glutamatergic transmission through NMDA receptors [47]. Cholinergic modulation of hippocampal astrocytes also leads to long-term inhibition of dentate granule cells through direct glial excitation of inhibitory interneurons [56]. In addition, cholinergic modulation of glial cell function has been observed in the retina [57] and the enteric nervous system [58]. Together, these studies demonstrate widespread actions of cholinergic signaling on glial activity states; however less is known about reciprocal signaling from astrocytes to cholinergic synapses. Our work in the peripheral nervous system suggests that these effects may be part of a broader regulatory system that includes glial control of their cholinergic inputs.

Satellite glia regulated multiple developmental processes in this study, including neuronal survival (Figs 7 and 8), cell body hypertrophy (Table 1) and cholinergic synapse formation (Fig 4). These are all promoted by NGF in developing sympathetic neurons [37, 59–61]. We showed that a neurotrophic factor, likely NGF, released by satellite glia partially supported the survival of the cultured sympathetic neurons. The production of neurotrophins by satellite glia is consistent with the reported expression of neurotrophins in other central and peripheral glial populations [62–66]. However, extensive evidence points to the central role of target-derived NGF produced by peripheral organs in the survival and morphological maturation of postnatal sympathetic neurons [43, 67]. Thus, our data suggest that glial-derived neurotrophic factors may provide a secondary source of neurotrophic signaling during postnatal development and in the mature the sympathetic circuit.

We previously showed synaptic modulation of sympathetic cholinergic transmission by neurotrophins [38], but further work will be needed to determine if glial-derived neurotrophins contribute to the synaptic effects of glial cells. We do not expect that glial-derived NGF is the primary source of neurotrophic signaling in this system, as NGF is retrogradely transported from peripheral targets *in vivo* [68]. It is interesting to speculate, however, that ganglionic sources of such factors could play a stabilizing role during development or following nerve injury. Peripheral nerve injury is accompanied by a reduction in NGF retrograde transport [69] and a dramatic decrease in sympathetic neuron activity and cholinergic synapses within the ganglia [70]. Thus, ganglionic sources of NGF could provide a compensatory source of neurotrophic signaling and would be consistent with activation of glia during pathological disruptions [71, 72].

The effects of satellite glia on sympathetic synaptic function suggest the potential for ongoing glial regulation in the sympathetic system. This is of particular interest in pathological situations, such as in cardiovascular disorders in which sympathetic over-activation is a common feature [19–21]. This idea is consistent with recently published work using selective activation of a glial-expressed Gq protein-coupled receptor in transgenic mice to show that acutely activated glial cells *in vivo* increased heart rate and cardiac output through the actions of the peripheral sympathetic system [30, 31]. This increase in heart rate was abolished by selective inhibition of peripheral glia activation, further establishing satellite glia as regulators of sympathetic-mediated cardiac function. The work described here demonstrates a link between ganglionic satellite glia and functional changes in the electrical properties of sympathetic neurons, providing a mechanistic model for the actions of satellite glia in driving heightened sympathetic tone and suggesting these glia as potential new targets to treat diseases of the peripheral organs.

## Supporting information

S1 Table (XLSX)

S1 Data (XLSX)

S2 Data (XLSX)

S3 Data (XLSX)

S4 Data (XLSX)

S5 Data (XLSX)

S6 Data (XLSX)

S7 Data (XLSX)
